# Beyond single scaffolding: a multidimensional scaffolding framework for flow-driven Game-Based Learning

**DOI:** 10.3389/fpsyg.2026.1950695

**Published:** 2026-10-05

**Authors:** Huei-Tse Hou

**Affiliations:** Graduate Institute of Applied Science and Technology, National Taiwan University of Science and Technology, Taipei, Taiwan

**Keywords:** flow, Game-Based Learning, learning performance, scaffolding, Zone of Proximal Development

## Abstract

Meta-analytic evidence indicates Game-Based Learning (GBL) to be beneficial for enhancing learning motivation and promoting knowledge construction, with the design and provision of scaffolding within games playing a critical role in enhancing learning outcomes. How to simultaneously foster learners’ flow and learning remains a significant challenge in GBL scaffolding design; insufficient or inappropriate scaffolding may negatively impact both the gaming experience and learning outcomes. Recent meta-analyses have found that GBL has a moderate-to-large effect on cognitive outcomes, but the effect sizes show high heterogeneity. Furthermore, no significant improvement was observed in metacognitive outcomes. This imbalance may stem from the limitations of GBL research approaches that focus primarily on a single type of scaffolding. As GBL is a highly interactive and complex learning process, research on multidimensional scaffolding mechanisms has garnered increasing attention in recent years for its potential to enhance GBL effectiveness; however, there is currently a lack of systematic theoretical foundations and design guidelines. This study is a perspective article. Based on recent empirical research on GBL scaffolding, this study proposes a theoretical framework for Multidimensional Scaffolding in Game-Based Learning (MDS-GBL) in accordance with the characteristics of multidimensional forms of Flow Theory and the Zone of Proximal Development (ZPD) and presents a practical framework for scaffolding design and assessment. This study also draws on the results of recent empirical research on multidimensional scaffolding to support the proposed theoretical framework and presents three design principles for multidimensional scaffolding to serve as a reference for researchers and designers.

## 1 Introduction

### 1.1 The role of scaffolding in Game-Based Learning

Meta-analytic evidence indicates that GBL effectively enhances academic achievement (Clark et al., 2016; Cai et al., 2022) and elicits positive affective responses and flow experiences (Hamari et al., 2016; Ngu et al., 2025). Furthermore, it can promote social interaction and cultivate learners’ problem-solving abilities (Clark et al., 2016; Hui and Mahmud, 2023). Scaffolding provides real-time guidance within games to enhance learning outcomes and flow. Providing sufficient or appropriately tailored scaffolding is a key factor in enabling learners to achieve both flow and deep learning simultaneously, and it constitutes a central issue in Game-Based Learning design.

### 1.2 Theoretical gaps in multidimensional skill gaps

Although the meta-analysis by Cai et al. (2022) found that GBL scaffolding effectively improves learning outcomes (*g* = 0.43) and also found high heterogeneity in effect sizes, this indicates that “how to design scaffolding” is far more critical than “whether to provide scaffolding.” A meta-analysis by Barz et al. (2024) found that GBL had a moderate-to-large effect on cognitive outcomes (*g* = 0.67) but did not significantly improve metacognitive outcomes. This imbalance may stem from the fact that most GBL designs focus primarily on scaffolding to support conceptual understanding, while potentially neglecting the configuration and in-depth exploration of scaffolding for dimensions such as procedural understanding, metacognition, and affective engagement. Existing soft/hard scaffolding frameworks have their limitations (Chen and Law, 2016) and may not fully address the guidance needs of GBL activities that are both interactive and complex. Currently, there is a relative lack of theoretical frameworks and design guidelines for multidimensional scaffolding. Therefore, this paper will establish a theoretical foundation for multidimensional scaffolding in GBL based on flow theory and the Zone of Proximal Development (ZPD) theory, identify core common principles, and propose a practical framework for multidimensional scaffolding design.

## 2 Theoretical foundations of the multidimensional GBL scaffolding theory

### 2.1 The connection between flow theory and GBL scaffolding

Unlike typical static learning activities, GBL activities feature relatively complex dynamic interaction mechanisms. Among these, flow is a key factor that reflects the learner’s level of cognitive focus and engagement in learning behaviors throughout the interactive learning process. Csikszentmihalyi’s (1990) theory of flow states that optimal experiences occur when there is a dynamic balance between the level of challenge in an activity and the individual’s skill level; if the challenge far exceeds skill, learners feel anxious, and if skill far exceeds the challenge, they feel bored. This study argues that in a GBL context, the core function of scaffolding lies in dynamically calibrating this challenge-skill balance, making it a decisive design factor in whether learners can enter and maintain a state of flow. The theoretical framework of this study is shown in Figure 1, and its core foundation is the following dynamic adjustment and calibration mechanism:

**Figure 1:**
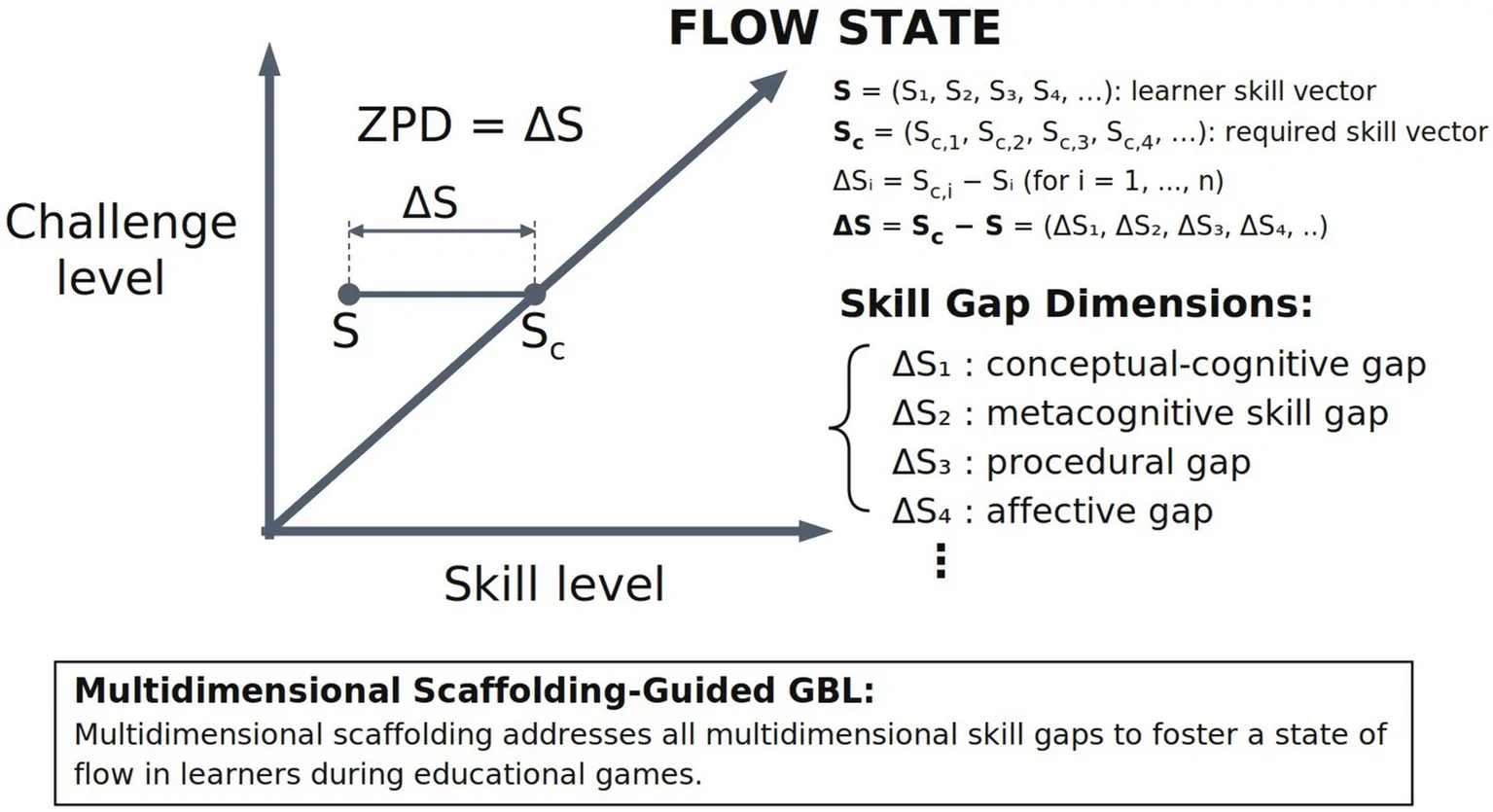
Relationship between multidimensional skill gaps and the state of flow.

*S_c_ represents a state of challenge-skill balance; when a player’s skill at a given time t is insufficient (S_t_ < S_c_), scaffolding is needed to bridge the gap ΔS, thereby performing dynamic calibration to achieve flow*.

### 2.2 The ZPD in complex game interaction contexts

Wood et al. (1976) operationalized Vygotsky’s (1978) Zone of Proximal Development (ZPD), defining scaffolding as the gap between what a learner can accomplish independently and what they can achieve with appropriate external support. In complex GBL environments, learners simultaneously face at least four types of ZPD phases: conceptual-cognitive gaps (cognitive load resulting from insufficient knowledge of the learning unit), metacognitive skill gap (lack of metacognitive skills), gaps in game rules understanding and procedural operations (unfamiliarity with game mechanics or task workflows), and gaps in affective engagement (maintaining motivation to tackle challenges, affective identification, or emotional immersion). Therefore, scaffolding designed for only one dimension inevitably fails to address the other dimensions—this is the fundamental assumption of the multidimensional scaffolding approach.

### 2.3 Formalized proposition of the multidimensional GBL ZPD: Δ*S*

Basawapatna et al. (2013) proposed the concept of the “Zone of Proximal Flow (ZPF)” in an attempt to integrate the ZPD with flow theory; however, they did not discuss how to apply it to the complex interactive mechanisms of GBL scaffolding. As shown in Figure 1, building on this foundation, this study defines the ZPD GBL in a GBL context as the following composite skill gap:


$$\mathrm{GBL}\;\mathrm{ZPD}=\mathrm{\Delta S}=S_{c}-S$$

$$S=(S_{1},S_{2},S_{3},S_{4},\ldots ):\text{learner skill vector}$$

$$S_{c}=(S_{c,1},S_{c,2},S_{c,3},S_{c,4},\ldots ):\text{required skill vector}$$

$$\Delta S_{i}=S_{c,i}-S_{i}\;(\text{for}\;i=1,\ldots ,n)$$

$$\mathrm{\Delta S}=S_{c}-S=(\Delta S_{1},\Delta S_{2},\Delta S_{3},\Delta S_{4},\ldots )$$

Here, S = (S_1_, S_2_, S_3_, S_4_, …) represents the learner’s current skill level, and S_c_ = (S_c,1_, S_c,2_, S_c,3_, S_c,4_, …) represents the skill level required for the challenging task. The composite gap ΔS can be decomposed into at least four independent dimensions: ΔS_1_ (conceptual-cognitive gap: knowledge gaps), ΔS_2_ (metacognitive skill gap: e.g., planning, monitoring, reflection, etc.), ΔS_3_ (procedural understanding gap: e.g., game mechanics and task flow), and ΔS_4_ (affective gap: e.g., motivation and emotions) and there may be additional dimensions of gaps that require further exploration and the development of corresponding scaffolding.

Since cognitive, metacognitive, procedural, and affective gaps are conceptually distinct and are not measured on the same scale, ΔS is represented as a multidimensional vector, ΔS = (ΔS_1_, ΔS_2_, ΔS_3_, ΔS_4_, …), rather than as the sum of the individual dimensions. It is important to note that the term “skill gap” used in this paper is an abbreviation for “multidimensional support-needs gap”; not every ΔS_i_ represents a skill in the strict psychological sense. For example, the affective dimension serves as a moderating factor rather than a skill. Each ΔS_i_ is measured using indicators appropriate to its domain (e.g., conceptual tests, metacognitive monitoring, task completion rates, motivation/emotion scales), standardized beforehand, and then compared. When consolidating metrics in game design practice to prioritize resource allocation, it is recommended to present them in a weighted standardized form: ΔS_agg_ = Σ w_i_·ΔS_i_^*^ (where ΔS_i_^*^ represents the standardized deviation and w_i_ are weights set according to practical design needs). However, it should be noted that the weighted form is intended solely as a reference for instructional design practice; it does not imply that the various dimensions can theoretically be directly added together on the same scale.

The core proposition of Multidimensional Scaffolding-based GBL (MDS-GBL) is:

*Multiple scaffolds should be deployed simultaneously within the game, along with collaborative mechanisms, to effectively cover the entire ΔS and provide optimal support for learners to maintain flow and maximize learning*.

This study argues that scaffolding supports flow through three parallel mechanisms: (i) enhancing learners’ effective skills, thereby reducing ΔS and achieving a balance between challenge and skill; (ii) reducing the perceived difficulty of the task; and (iii) supporting emotional regulation. Among these, the emotional dimension is viewed as a moderating factor that shapes the perception of the challenge-skill balance. It is worth noting that the benefits of multidimensional scaffolding do not stem from simply stacking more scaffolds but depend on whether the scaffolds precisely align with each ΔS_i_ dimension.

### 2.4 The MDS-GBL framework

Based on the ΔS multidimensional propositions shown in Figure 1, the practical design framework of MDS-GBL addresses the fundamental elements of gamified learning identified by Prensky (2007)—including learning objectives (knowledge acquisition), problem-solving (strategic reflection), rules (understanding of interaction rules), and entertainment and enjoyment (emotional motivation and engagement)—by mapping these functional dimensions to the design of various types of scaffolding. Each dimension corresponds to a ΔS_i_ component (Figure 2). The core design mechanism of the framework lies in the following:

**Figure 2:**
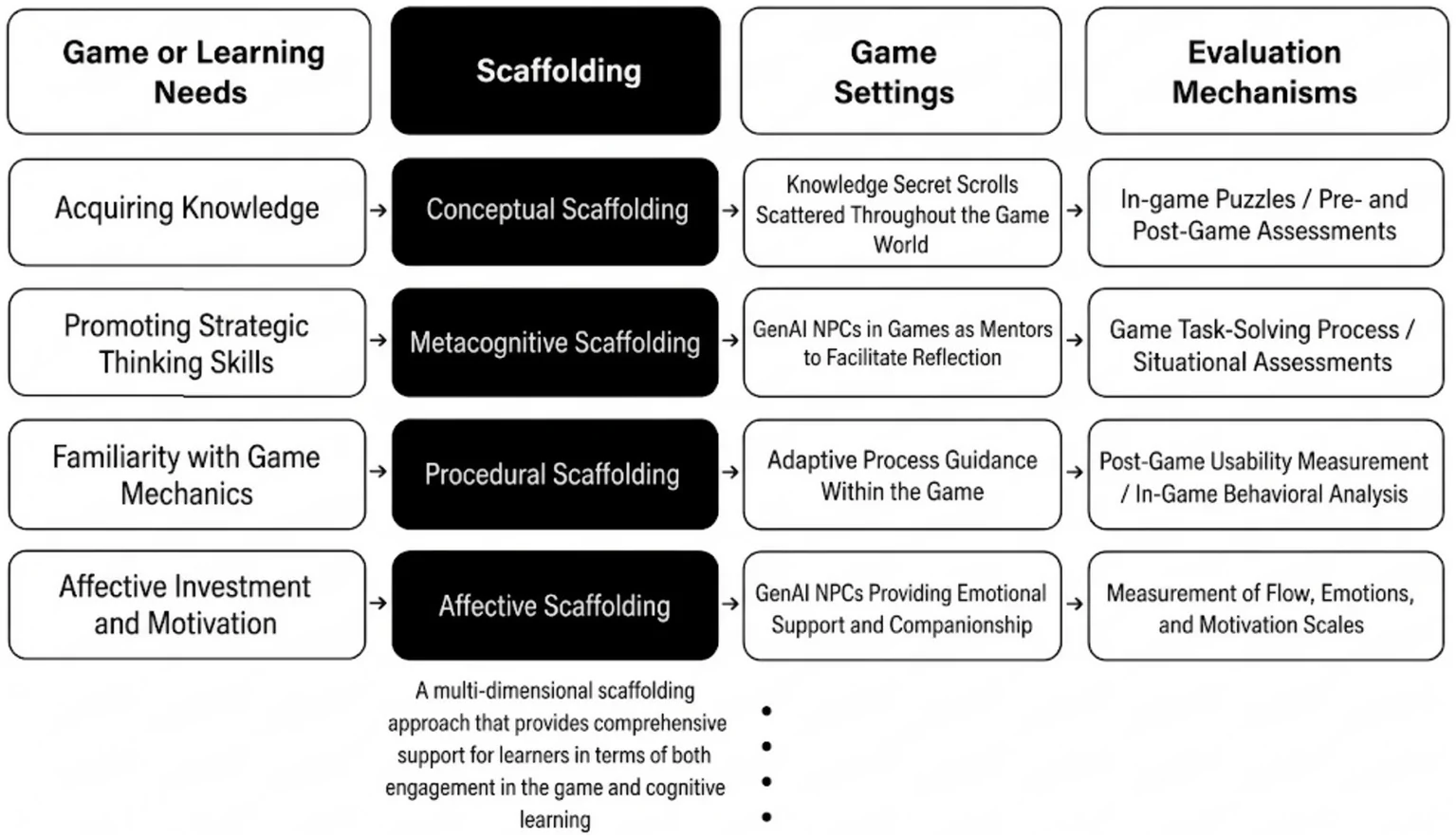
MDS-GBL design framework.

*The synergistic deployment of multidimensional scaffolding addresses all learning objectives or game requirements, and the types of scaffolding can be dynamically expanded as new ΔS_i_ dimensions are discovered or identified.*

Furthermore, although each framework has a primary target dimension, it may produce cross-dimensional effects; a multidimensional framework mechanism can provide an environment for coordinating these primary and cross-dimensional effects, enabling them to collectively cover the entire ΔS.

## 3 Empirical studies of multidimensional scaffolding

Multiple studies are consistent with the view that the combined effect of deploying multiple types of scaffolding in tandem is superior to the independent use of a single type. A meta-analysis by Belland et al. (2015) found that in complex and unstructured real-world learning contexts, support involving multidimensional scaffolding must be provided simultaneously. A meta-analysis by Barz et al. (2024) revealed that GBL has no significant effect on metacognitive outcomes, this motivates the design of the MDS-GBL framework. Current GBL scaffolding designs tend to focus on either a single type of scaffold or on various types of scaffolds used independently. Under this design approach, there is a lack of metacognitive scaffolding to promote higher-order thinking, as well as a lack of synergistic mechanisms where metacognitive scaffolding complements existing cognitive scaffolding. For example, Chernikova et al. (2020) found that the synergistic combination of scaffolding that promotes reflection (metacognitive scaffolding) and scaffolding that provides demonstrations (procedural scaffolding) helps enhance learning outcomes. A meta-analysis by Cai et al. (2022) also revealed that the effectiveness of GBL scaffolding is highly heterogeneous, which underscores the importance of how scaffolding is selected and designed, as well as whether the functions of various types of scaffolding can truly fulfill their supportive role. In terms of the ΔS framework, these findings are consistent with the notion that the skill gaps learners face in the GBL process are multidimensional; a single type of scaffolding primarily addresses a specific ΔS_i_, while potentially exerting secondary cross-dimensional effects, and comprehensive coverage of ΔS relies on the appropriate deployment and synergy of multidimensional scaffolding.

Kuo and Hou’s (2025) study on collaborative decision-making in GBL provided behavioral-level supportive evidence through lag sequence analysis (LSA): when cognitive, peer, and metacognitive scaffolds operate synergistically, learners’ interactive behaviors shift toward deeper, higher-order thinking and strategic deliberation; this shift is more difficult to achieve under conditions where only cognitive scaffolding is present; this evidence is consistent with the synergistic nature of multidimensional scaffolding in eliciting higher-order cognitive behaviors. Various synergistic effects of scaffolding have also been supported in meta-analyses of different learning contexts (Chernikova et al., 2020).

Furthermore, the method of scaffolding integration is a critical factor, and the effectiveness of contextual scaffolding embedded within game contexts is receiving increasing attention and discussion. Generative AI (Gen AI)—which features natural language dialogue and is well-suited to presenting various contextual scenarios within games—serves as an effective mechanism for delivering scaffolding. Gong et al. (2026) compared GenAI scaffolding with traditional static scaffolding and found that the former reported advantages in both learning outcomes and self-regulated learning behaviors. Many studies (e.g., Chen and Hou, 2024; Ngu et al., 2025; Chien et al., 2025) have also highlighted the advantages of using GenAI non-player characters (NPCs) for scaffolding in GBL. These characters can be seamlessly integrated into scenarios through role-playing, maintaining high levels of flow, promoting learning, and enhancing situational realism. Furthermore, presented as NPCs, they can simultaneously provide cognitive guidance and affective support. This technology has the potential to synchronously address ΔS_1_, ΔS_2_, ΔS_3_ and ΔS_4_ based on the learner’s real-time state, thereby extending the application effectiveness of the aforementioned framework. These types of NPCs operate on a single mechanism and have cross-dimensional effects (they have a primary objective dimension, such as cognitive guidance, and also produce secondary effects, such as affective support or metacognitive guidance).

However, the use of GenAI NPCs to provide real-time cognitive guidance and emotional support must also take ethical considerations into account. The real-time analysis of data collected from learners to infer their behavior and emotions involves sensitive issues such as informed consent and emotional profiling; it is recommended that this be conducted in a transparent manner that allows learners to opt out. At the same time, content generated by GenAI may result in biased feedback, necessitating content moderation and teacher supervision. Therefore, the personalized assessment and GenAI support within this framework should be implemented within clear ethical boundaries, including data protection, bias monitoring, and preserving decision-making authority for teachers and students.

## 4 Discussion

### 4.1 Multidimensional scaffolding design principles for GBL

Based on the theoretical framework and three findings discussed above, and in conjunction with the design and assessment framework shown in Figure 2, this paper proposes three multidimensional scaffolding design principles for GBL:

(1) Diagnose before design: use the ΔS_i_ gap diagnosis as the starting point for scaffolding planning

A meta-analysis by Belland et al. (2015) pointed out that scaffolding design lacking a preliminary needs evaluation is one of the primary causes of poor outcomes in scaffolding interventions. Scaffolding design should begin by assessing the extent of the target learners’ gaps across the four dimensions of ΔS_1_, ΔS_2_, ΔS_3_ and ΔS_4_, using this as the basis for selecting the type of scaffolding (corresponding to the first column of Figure 2, “Game or Learning Needs”). Different learning and game needs correspond to different weights or priorities for ΔS_i_: those focused primarily on knowledge acquisition should prioritize addressing ΔS_1_; those requiring a high level of player engagement should prioritize ensuring coverage of ΔS_4_. Furthermore, learners with different cognitive styles respond differently to various scaffolding types (Chang and Yang, 2023), which means that the diagnosis of ΔS_i_ must not only identify the dimensions of the gaps but also account for individual differences among learners.

In practice, the game system can infer these dimensions by detecting and observing learner behavior: detecting conceptual mistakes indicates a ΔS₁ (conceptual/cognitive) gap; detecting insufficient monitoring or reflective behavior indicates a ΔS_2_ (metacognitive) gap; detecting repeated failures to execute game procedures indicates a ΔS_3_ (procedural) gap; detecting low learner engagement or declining motivation can indicate a ΔS_4_ (affective) gap in engagement.

(2) Embed and synergize: achieving contextual embedding and synergistic deployment of multidimensional scaffolding

An effective GBL scaffolding framework should not function as a hint system separate from the game but should be naturally embedded within the game’s interactive mechanisms and contextual framework (corresponding to the third column, “Game Settings,” in Figure 2), ensuring that multiple scaffolding types form synergistic relationships rather than merely existing in parallel. The study by Kuo and Hou (2025) confirms the complementary and synergistic nature of the various types of scaffolding. A key design checkpoint for synergy is whether the effectiveness of the other scaffolding components also decreases when any single one is removed; if not, these scaffolding components may merely be parallel rather than truly synergistic. In terms of validation, the synergistic effect can be tested as a statistically significant interaction effect in a factorial design (e.g., a 2 × 2 factorial design combining the presence or absence of metacognitive scaffolding with the presence or absence of procedural scaffolding) to determine whether the combined effect differs from what would be expected under an additive model.

(3) Establish a dynamic diagnostic mechanism: maintain the flow channel and facilitate scaffold fading

Achieving flow requires that the scaffold be capable of responding in real time to the challenge-skill gap (corresponding to the real-time diagnostic function in the “Evaluation Mechanism” column, fourth column of Figure 2). Designers should establish dynamic diagnostic mechanisms—such as GenAI NPC feedback (Chien et al., 2025), real-time behavioral monitoring indicators (Faber et al., 2023), or formative diagnostic scaffolding procedures (Lin and Hou, 2023). When the ΔSi gap exceeds a threshold, scaffolding corresponding to that category should be provided to prevent learners from slipping into the anxiety or boredom zones. The core advantage of dynamic diagnostic mechanisms lies in their ability to allocate support across dimensions based on the learner’s real-time state, whereas static scaffolding is typically oriented toward a single, predefined ΔS_i_. Therefore, the design of assessment mechanisms should be planned in tandem with scaffolding design, rather than added as an afterthought. Furthermore, through real-time diagnosis, the density of actively provided scaffolding should gradually decrease as the learner progresses through stages. By operationalizing the “scaffolding fading” concept proposed by Wood et al. (1976), learners can be guided to transition from reliance on support to autonomous learning. Furthermore, in the post-game evaluation of multidimensional scaffolding, measuring players’ perceptions of the usefulness of individual scaffolds within the same game is an important reference indicator (e.g., Chien et al., 2025) that can inform adjustments to the scaffolding in future game versions.

This leads to two design considerations. First, the threshold for triggering scaffolding can be established using norm-referenced (peer percentile), criterion-referenced (preset proficiency threshold), or data-driven (real-time behavioral sequence detection) methods, and should be calibrated based on task difficulty and individual differences. Second, there are also boundary conditions regarding how scaffolding promotes flow: excessive or premature scaffolding may disrupt immersion, increase external cognitive load, or reduce the challenge to the point of boredom, which are aspects that require careful consideration in the design process.

### 4.2 Conclusion and future study

To promote educational games that simultaneously achieve flow and deep learning, this paper takes the multidimensional vector formulation ΔS = (ΔS_1_, ΔS_2_, ΔS_3_, ΔS_4_, …) as its theoretical core and, drawing on the multidimensional synergistic scaffolding effect (Belland et al., 2015; Kuo and Hou, 2025) and contextual scaffolding embedding (Chen and Hou, 2024) as its empirical foundation, proposes the MDS-GBL framework and three design principles.

As a perspective article, this paper focuses on deriving a theoretical framework and design principles through literature review; future research should systematically validate the predictive validity of the framework through controlled experiments. Furthermore, technological advancements in generative AI scaffolding (Chien et al., 2025; Gong et al., 2026) have made the design of dynamic diagnostics more feasible at the engineering level: normalizing the intervention logic of AI NPCs based on ΔSi diagnostic results (e.g., triggering ΔS_1_ support when conceptual difficulties arise, or triggering ΔS₄ affective interventions when motivation declines) will be the key pathway for adaptive diagnostics within the MDS-GBL framework. In this regard, utilizing the analysis of multimodal learning process data for diagnostic purposes holds great potential (Azevedo et al., 2025).

Finally, to ensure that the framework can be empirically verified in the future, this study proposes four propositions for future research to test: (1) Multidimensional scaffolding that is matched (arranged based on ΔSᵢ diagnosis) is superior to mismatched scaffolding; (2) When learner needs change over time, adaptive scaffolding is superior to fixed scaffolding; (3) Combinations of scaffolds produce interaction effects beyond an additive model; (4) Scaffolding fade-out is more effective than continuous support in enhancing subsequent autonomous learning performance.

## Data Availability

The original contributions presented in the study are included in the article/supplementary material, further inquiries can be directed to the corresponding author.

## References

[ref1] Azevedo R. Dever D. Wiedbusch M. Brosnihan A. Delgado T. Marano C. . (2025). “A taxonomy for enhancing metacognitive adaptivity and personalization in serious games using multimodal trace data,” in Serious Games, eds. Plass J. L. Ochoa X. (Cham, Switzerland: Springer), 27–40.

[ref2] Barz N. Benick M. Dörrenbächer-Ulrich L. Perels F. (2024). The effect of digital game-based learning interventions on cognitive, metacognitive, and affective-motivational learning outcomes in school: a meta-analysis. Rev. Educ. Res. 94, 193–227. doi: 10.3102/00346543231167795

[ref3] Basawapatna A. R. Repenning A. Koh K. H. Nickerson H. (2013). The zones of proximal flow: guiding students through a space of computational thinking skills and challenges. Proceedings of ICER'13, 67–74.

[ref4] Belland B. R. Walker A. E. Olsen M. W. Leary H. (2015). A pilot meta-analysis of computer-based scaffolding in STEM education. J. Educ. Technol. Soc. 18, 183–197.

[ref5] Cai Z. Mao P. Wang D. He J. Chen X. Fan X. (2022). Effects of scaffolding in digital game-based learning on student's achievement: a three-level meta-analysis. Educ. Psychol. Rev. 34, 537–574. doi: 10.1007/s10648-021-09655-0

[ref6] Chang C.-C. Yang S.-T. (2023). Interactive effects of scaffolding digital game-based learning and cognitive style on adult learners' emotion, cognitive load and learning performance. Int. J. Educ. Technol. High. Educ. 20:16. doi: 10.1186/s41239-023-00385-7

[ref7] Chen Y. C. Hou H. T. (2024). A mobile contextualized educational game framework with ChatGPT interactive scaffolding for employee ethics training. J. Educ. Comput. Res. 62, 1737–1762. doi: 10.1177/07356331241268505

[ref8] Chen C. H. Law V. (2016). Scaffolding individual and collaborative game-based learning in learning performance and intrinsic motivation. Comput. Hum. Behav. 55, 1201–1212. doi: 10.1016/j.chb.2015.03.010

[ref9] Chernikova O. Heitzmann N. Stadler M. Holzberger D. Seidel T. Fischer F. (2020). Simulation-based learning in higher education: a meta-analysis. Rev. Educ. Res. 90, 499–541. doi: 10.3102/0034654320933544

[ref10] Chien C.-C. Chan H.-Y. Hou H.-T. (2025). Learning by playing with generative AI: design and evaluation of a role-playing educational game with generative AI as scaffolding for instant feedback interaction. J. Res. Technol. Educ. 57, 894–913. doi: 10.1080/15391523.2024.2338085

[ref11] Clark D. B. Tanner-Smith E. E. Killingsworth S. S. (2016). Digital games, design, and learning: a systematic review and meta-analysis. Rev. Educ. Res. 86, 79–122. doi: 10.3102/0034654315582065, 26937054 PMC4748544

[ref12] Csikszentmihalyi M. (1990). Flow: The Psychology of Optimal Experience. New York: Harper & Row.

[ref13] Faber T. J. E. Dankbaar M. E. W. Kickert R. van den Broek W. W. van Merriënboer J. J. G. (2023). Identifying indicators to guide adaptive scaffolding in games. Learn. Instr. 83:101666. doi: 10.1016/j.learninstruc.2022.101666, 42574925

[ref14] Gong Y. Wang M. He L. Xu C. Yu Y. (2026). Asking, playing, learning: investigating large language model-based scaffolding in digital game-based learning for elementary artificial intelligence education. J. Educ. Comput. Res. 64, 311–343. doi: 10.1177/07356331251396354

[ref15] Hamari J. Shernoff D. J. Rowe E. Coller B. Asbell-Clarke J. Edwards T. (2016). Challenging games help students learn: an empirical study on engagement, flow and immersion in game-based learning. Comput. Hum. Behav. 54, 170–179. doi: 10.1016/j.chb.2015.07.045

[ref16] Hui H. B. Mahmud M. S. (2023). Influence of game-based learning in mathematics education on the students' cognitive and affective domain: a systematic review. Front. Psychol. 14:1105806. doi: 10.3389/fpsyg.2023.1105806, 37057144 PMC10086333

[ref17] Kuo C. C. Hou H. T. (2025). Game-based collaborative decision-making training: a framework and behavior analysis for a remote collaborative decision-making skill training game using multidimensional scaffolding. Univ. Access Inf. Soc. 24, 867–883. doi: 10.1007/s10209-024-01103-4

[ref18] Lin Y. C. Hou H. T. (2023). Multi-dimensional evaluation of an educational board game using real-time diagnostic procedure scaffolding: analysis of learners’ learning effectiveness, flow, anxiety, and emotion. Educ. Psychol. 43, 874–894. doi: 10.1080/01443410.2023.2259139

[ref19] Ngu P. C. Chien C. C. Ho Y. T. Hou H. T. (2025). A generative AI educational game framework with multi-scaffolding supports workplace competency development. Comput. Educ. 239:105421. doi: 10.1016/j.compedu.2025.105421, 42574925

[ref20] Prensky M. (2007). Digital Game-Based Learning. St. Paul, MN: Paragon House.

[ref21] Vygotsky L. S. (1978). Mind in Society: The Development of Higher Psychological Processes. Cambridge, MA: Harvard University Press.

[ref22] Wood D. Bruner J. S. Ross G. (1976). The role of tutoring in problem solving. J. Child Psychol. Psychiatry 17, 89–100. doi: 10.1111/j.1469-7610.1976.tb00381.x, 932126

